# Validation of a Metagenomic Next-Generation Sequencing Assay for Lower Respiratory Pathogen Detection

**DOI:** 10.1128/spectrum.03812-22

**Published:** 2022-12-12

**Authors:** Zhenli Diao, Huiying Lai, Dongsheng Han, Bin Yang, Rui Zhang, Jinming Li

**Affiliations:** a National Center for Clinical Laboratories, Institute of Geriatric Medicine, Chinese Academy of Medical Sciences, Beijing Hospital/National Center of Gerontology, Beijing, People’s Republic of China; b National Center for Clinical Laboratories, Chinese Academy of Medical Sciences & Peking Union Medical College, Beijing, People’s Republic of China; c Beijing Engineering Research Center of Laboratory Medicine, Beijing, People’s Republic of China; d Department of Laboratory Medicine, Beijing Hospital, National Center of Gerontology Institute of Geriatric Medicine, Chinese Academy of Medical Sciences, Beijing, People’s Republic of China; e Key Laboratory of Clinical In Vitro Diagnostic Techniques of Zhejiang Province, Department of Clinical Laboratory, First Affiliated Hospital, College of Medicine, Zhejiang University, Hangzhou, People’s Republic of China; f Vision Medicals Center for Infectious Diseases, Guangzhou, People’s Republic of China; University Paris-Saclay, AP-HP Hôpital Antoine Béclère, Service de Microbiologie, Institute for Integrative Biology of the Cell (I2BC), CEA, CNRS

**Keywords:** mNGS, validation, metagenomics, respiratory tract infection, pneumonia

## Abstract

Lower respiratory infection (LRI) is the most fatal communicable disease, with only a few pathogens identified. Metagenomic next-generation sequencing (mNGS), as an unbiased, hypothesis-free, and culture-independent method, theoretically enables the detection of all pathogens in a single test. In this study, we developed and validated a DNA-based mNGS method for the diagnosis of LRIs from bronchoalveolar lavage fluid (BALF). We prepared simulated *in silico* data sets and published raw data sets from patients to evaluate the performance of our in-house bioinformatics pipeline and compared it with the popular metagenomics pipeline Kraken2-Bracken. In addition, a series of biological microbial communities were used to comprehensively validate the performance of our mNGS assay. Sixty-nine clinical BALF samples were used for clinical validation to determine the accuracy. The in-house bioinformatics pipeline validation showed a recall of 88.03%, precision of 99.14%, and F1 score of 92.26% via single-genome simulated data. Mock *in silico* microbial community and clinical metagenomic data showed that the in-house pipeline has a stricter cutoff value than Kraken2-Bracken, which could prevent false-positive detection by the bioinformatics pipeline. The validation for the whole mNGS pipeline revealed that overwhelming human DNA, long-term storage at 4°C, and repeated freezing-thawing reduced the analytical sensitivity of the assay. The mNGS assay showed a sensitivity of 95.18% and specificity of 91.30% for pathogen detection from BALF samples. This study comprehensively demonstrated the analytical performance of this laboratory-developed mNGS assay for pathogen detection from BALF, which contributed to the standardization of this technology.

**IMPORTANCE** To our knowledge, this study is the first to comprehensively validate the mNGS assay for the diagnosis of LRIs from BALF. This study exhibited a ready-made example for clinical laboratories to prepare reference materials and develop comprehensive validation schemes for their in-house mNGS assays, which would accelerate the standardization of mNGS testing.

## INTRODUCTION

Lower respiratory infections (LRIs) were responsible for approximately 2.6 million deaths worldwide in 2019 and remain the world’s deadliest communicable disease (1). A 2015 large-scale population-based surveillance study reported that the pathogen was detected in only 38% of adults with community-acquired pneumonia, with viruses identified in 27% and bacteria in 14% (2). Early and accurate microbiological diagnoses contribute to the adoption of tailored antibiotic therapy and reduction of mortality, but such diagnoses are often challenging due to the inherent shortcomings of current microbiological tests. Conventional culture-dependent methods, whose accuracy is affected by the early administration of broad-spectrum antimicrobial drugs, have a long culture period and low sensitivity. Microscopy matrix-assisted laser desorption ionization–time of flight mass spectrometry can accurately identify bacteria and fungi within seconds to minutes, but it depends on isolation from pure cultures (3). Serology and PCR are culture-independent and convenient methods; however, prior knowledge of the suspected pathogens is necessary. Targeted sequencing is sensitive for the detection of selected organism types, but limitations in the breadth of detected pathogens hinder its ability to capture the overall perspective of the pathogen spectrum and discover novel pathogens.

Currently, metagenomic next-generation sequencing (mNGS) has carved out a space in diagnosing infectious diseases owing to its advantages of being culture independent, unbiased, and hypothesis free (3). Theoretically, mNGS enables the detection of all pathogens in a single test, including some microbes that are usually missed by traditional detection methods (such as fastidious microbes or novel pathogens) (4). These unique advantages of mNGS accelerate its translation from bench to bedside. An increasing number of clinical and independent third-party testing laboratories have developed in-house mNGS tests for the diagnosis of complicated infectious cases, all of which are considered laboratory-developed tests (LDTs). The Clinical Laboratory Improvement Amendments required laboratories to fully validate the LDTs and established the performance characteristics before implementation in the clinical setting (5, 6). Performance specifications for LDTs must be established, including accuracy, precision, reportable range, reference interval, analytical sensitivity, and analytical specificity (6). Several outstanding examples of mNGS validation strategies are available. For example, in 2019, Miller and colleagues developed and validated the performance of a clinical mNGS assay for pathogen detection in cerebrospinal fluid (CSF) samples (7). Blauwkamp et al. described the analytical and clinical validation of a microbial cell-free DNA-based mNGS test to aid in the diagnosis of infectious disease in the same year (8). However, it is more challenging to identify the pathogen in respiratory tract infections than in sterile site infections, as the respiratory tract is colonized by commensal oral flora organisms. In addition, the overwhelming amount of host nucleic acids in respiratory specimens (up to ~95% in nasopharyngeal aspirate samples) increases the sequencing costs and decreases the analytical sensitivity (9).

This report describes the development and validation of a DNA-based mNGS protocol for the diagnosis of LRIs in bronchoalveolar lavage fluid (BALF) samples. We evaluated the performance of the in-house bioinformatics pipeline and compared it with that of the Kraken2-Bracken pipeline. In addition, we prepared biological samples and designed a comprehensive validation scheme to assess the analytical performance of our mNGS assay, including the limits of detection (LoD), linearity, precision, interference, stability, and accuracy.

## RESULTS

### Study design and overview.

An mNGS method was developed for pathogen detection from BALF samples, which included host depletion, nucleic acid extraction, library preparation, sequencing, and bioinformatics analysis. Up to 20 libraries were processed in parallel in each sequencing run, with an overall sample-to-answer time of approximately 24 h.

**FIG 1:**
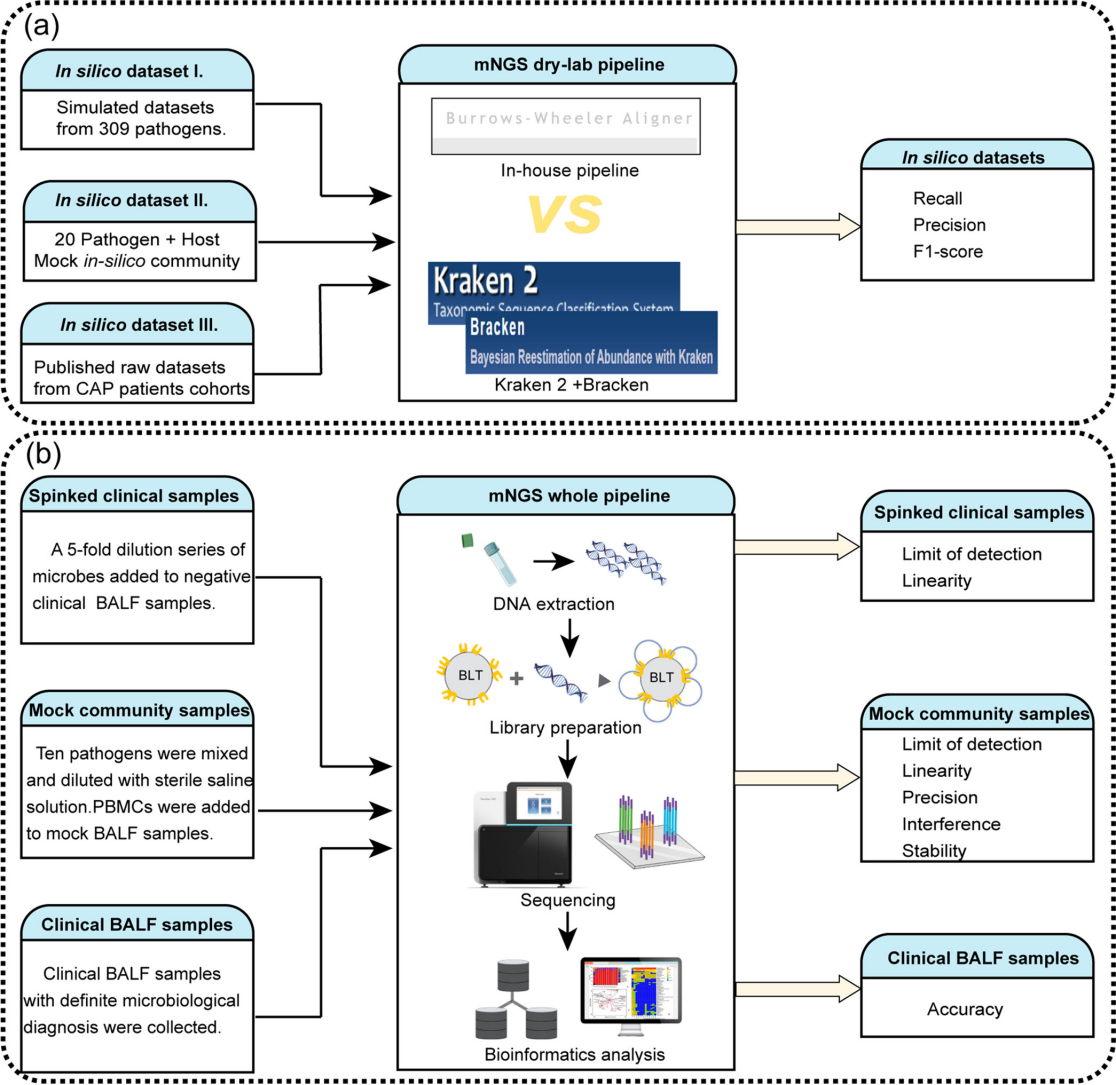
Validation study design. (a) Bioinformatics pipeline validation design; (b) whole metagenomic next-generation sequencing (mNGS) pipeline validation design. BALF, bronchoalveolar lavage fluid; BLT, bead-linked transposomes; CAP, community-acquired pneumonia; PBMCs, peripheral blood mononuclear cells.

We validated the performance of the mNGS assay at two levels: bioinformatics pipeline validation and whole mNGS pipeline validation (Fig. 1). For “dry-lab” validation, we used simulated *in silico* data sets and published raw data sets from patients to validate the in-house bioinformatics pipeline and compared it with the popular metagenomics pipeline Kraken2-Bracken. Three types of biological specimens were included for the whole mNGS pipeline validation: spiked clinical BALF samples, mock microbial community samples, and clinical BALF samples with definite microbiological diagnosis. A series of spiked clinical samples were used to evaluate the LoD and linearity of the mNGS assay. A set of designed mock microbial community samples containing 10 representative pathogens was utilized to comprehensively evaluate the analytical performance (LoD, linearity, precision, interference, and stability). Sixty-nine clinical BALF samples with definite microbiological diagnoses were tested to assess the accuracy of mNGS compared with that of culture or composite reference standards.

A list of background microorganisms from the reagents, the centrifuge, and the biosafety cabinet was generated (see Fig. S1 and Table S1 in the supplemental material), and the top three background microorganisms were Acinetobacter schindleri, Propionibacterium acnes, and Acinetobacter johnsonii. The calculated thresholds and plotted receiver operating characteristic (ROC) curves for 10 representative pathogens of LRIs enrolled in this study are available in Table S2 and Fig. S2, respectively. These unique thresholds showed excellent classification performance, with an average area under the ROC curve (AUC) of up to 0. 961 (95% confidence interval [CI], 0.933 to 0.989).

### Metagenomic bioinformatics validation.

**FIG 2:**
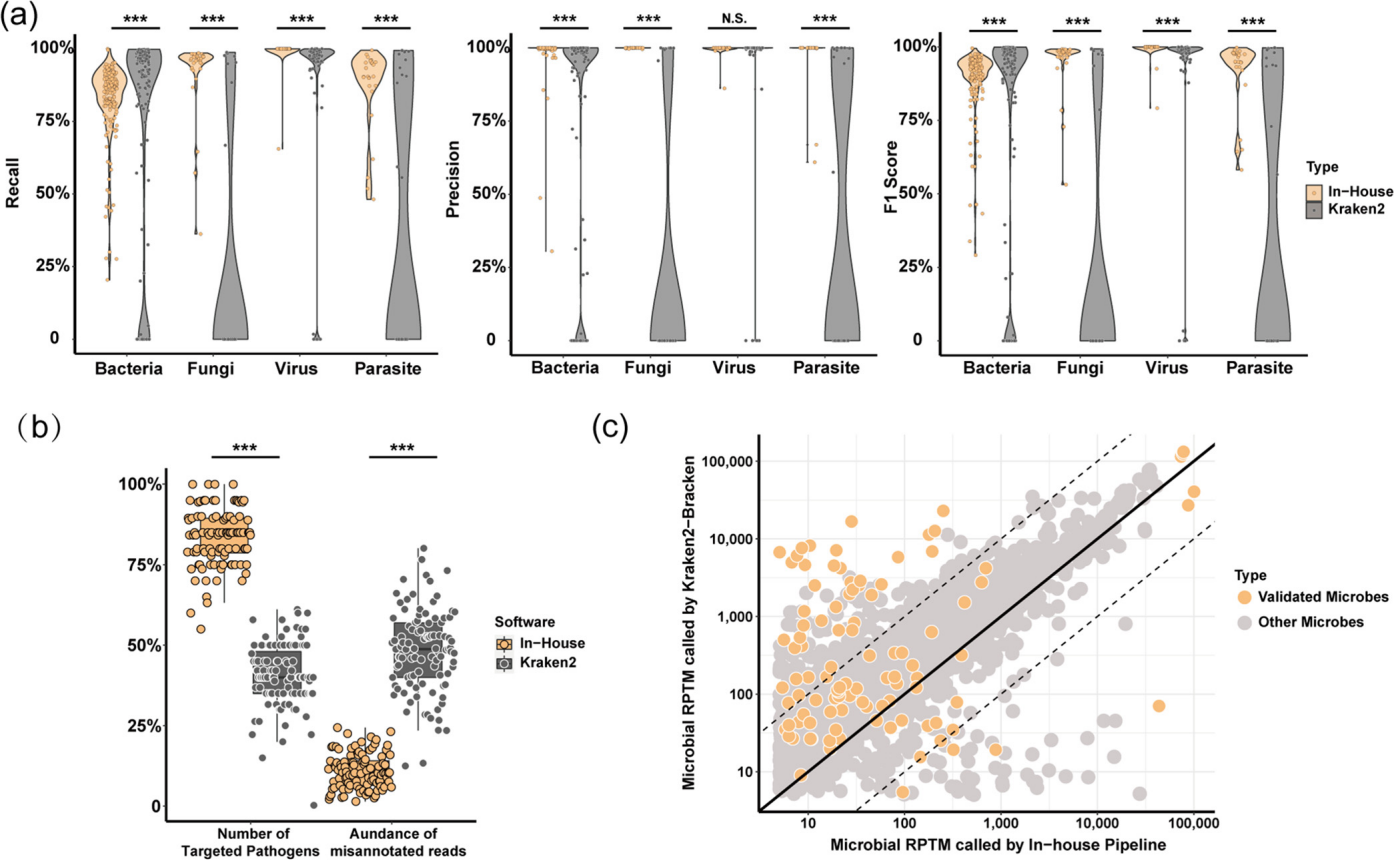
Bioinformatics performance of the in-house mNGS pipeline and Kraken2-Bracken pipeline. (a) Performance of our in-house mNGS pipeline and Kraken2-Bracken pipeline when analyzing single-genome simulated data. (b) Number of recalled targeted microbes and abundance of nonspecific microbes generated by the two pipelines when analyzing mixed microbial simulation data. (c) Observed number of mapping reads for identical microbes between the in-house mNGS pipeline and Kraken2-Bracken pipeline when analyzing published clinical data sets. N.S., not significant. ***, *P* < 0.001 by Wilcoxon test.

To exclude false detection by bioinformatics analysis, we first evaluated our in-house bioinformatics pipeline for metagenomic analysis by comparing the popular metagenomic methods Kraken2 and Bracken (10). We prepared three data sets for dry-lab validation at different levels (see Materials and Methods). In short, we first simulated single-genome data sets *in silico* from 309 species that are well-known human pathogens as data set I. Second, we generated 100 mock microbial samples with 20 pathogen and host reads as data set II, and third, we involved clinical data from a public cohort as data set III (11). Then, we calculated the recall, precision, and F1 score of each species detected by in-house and Kraken2-Bracken. Our in-house bioinformatics pipeline showed a recall of 88.03% (95% CI, 73.90% to 100.00%), precision of 99.14% (95% CI, 93.28% to 100.00%), and F1 score of 92.26% (95% CI, 73.91% to 100.00%) for the single-genome simulated data from 309 pathogens. There were 18.71% (32/171) bacteria, 78.79% (26/3) fungi, 6.41% (5/78) viruses, and 59.26% (16/27) parasites with no correct read assigned by the Kraken2-Bracken pipeline and its largest public database (see Materials and Methods). However, all pathogens could be recalled by the in-house pipeline (Fig. 2a and Table S3), which implies that false negatives will be involved in unsuitable databases. We also found that there were significant differences in the precision of different types of pathogens between the two bioinformatics pipelines (Fig. 2a) (Wilcoxon test, *P* value < 0.001). In addition, the in-house bioinformatics pipeline showed better specificity for the 10 representative pathogens of LRIs and a lower recall rate than Kraken2-Bracken (Fig. S3).

In data set II, the proportion of recalled targeted pathogens was higher for the in-house pipeline (in-house pipeline, 83.35% [95% CI, 74.45% to 92.25%]; Kraken2, 40.63% [95% CI, 30.54% to 50.72%], Wilcoxon test, *P* value < 0.001) (Fig. 2b). The abundance of misannotated reads was higher via the Kraken2-Bracken pipeline (in-house pipeline, 10.94% [95% CI, 5.51% to 16.37%]; Kraken2, 48.02% [95% CI, 35.08% to 60/96%], Wilcoxon test, *P* value < 0.001) (Fig. 2b). In data set III, we used a published severe community-acquired pneumonia patient cohort to evaluate whether the microbial abundances of the two different bioinformatics pipelines were comparable (11). We detected all nine validated pathogens in this study (human adenoviruses [HAdVs] were excluded because there was a lack of adenovirus pneumonia patients in the published cohort) (Fig. 2c, orange). A total of 3.88% (4/103) of validated microbes were detected at tenfold relative abundance higher by the in-house pipeline than by Kraken2, and 40.78% (42/103) were detected at tenfold relative abundance higher by Kraken2 than by the in-house pipeline (Fig. 2c). The results showed that Kraken2 preferred a higher read assignment either in a single-genome data set or in a clinical data set, which might cause bioinformatics false positives. These results indicated that the in-house pipeline has a strict cutoff value of read alignment/annotation compared with Kraken2-Bracken.

### Performance of the in-house mNGS assay.

The LoD and linearity of mNGS were evaluated by spiked clinical BALF samples and mock microbial community samples, respectively. Serial dilutions of Streptococcus pneumoniae and Pseudomonas aeruginosa (from 10,000 to 32 copies/mL) were added to a mix of two pooled negative BALF samples, with four replicates at each dilution (Table S4). Mock microbial community samples were prepared by spiking 10 representative pathogens of LRIs into the simulated BALF matrix (normal saline containing 10^5^ peripheral blood mononuclear cells [PBMCs]/mL) in a series of 5-fold dilutions from 1:1 (no dilution) to 1:78,125 with four replicates (Table 1 and Table S4). The LoD for each organism was calculated using probit regression analysis as the concentration at which mNGS testing successfully detected the pathogens in 95% of replicates (7). The LoD calculated by mock microbial community samples were 17.8 to 791.1 copies/mL for bacteria, 327.6 copies/mL for filamentous fungi, 2.4 copies/mL for yeast, and 1,191.2 copies/mL for DNA viruses (Table 2). The LoD generated by spiked clinical BALF samples for S. pneumoniae was 682.75 copies/mL and that for P. aeruginosa was 676.04 copies/mL, which were higher than those assessed by mock community samples (631.29 copies/mL for S. pneumoniae and 127.59 copies/mL for P. aeruginosa). However, it was noteworthy that the LoD calculated from different sample types varied on the same order of magnitude. A strong linear correlation between the log_10_-transformed total input organism load (copies per milliliter) and the log_10_-transformed numbers of mapped reads per 20 million (RPTM) was observed both in spiked clinical BALF samples and mock microbial community samples (linear regression *R*^2^ = 0.87 to 0.97) (Fig. S4). Therefore, facing the reality that sufficient, uniform, and negative BALF samples are difficult to acquire, it is feasible to evaluate the mNGS performance using simulated microbial community samples.

**TABLE 1:** Efficient and simple test plan for analytical validation among mock microbial community samples

Parameter	Presence at indicated dilution ratio							
	1:1	1:5	1:25	1:125	1:625	1:3,125	1:15,625	1:78,125
LoD^*a*^	Χ	Χ	Χ	Χ	Χ	Χ	Χ	Χ
Linearity	Χ	Χ	Χ	Χ	Χ	Χ	Χ	Χ
Precision				Χ				
Interference			Χ					
Stability			Χ					

a LoD, limit of detection.

**TABLE 2:** LoD and precision of our in-house mNGS assay^*a*^

Organism	Type	LoD (copies/mL)	Quantitative precision (CV), %	
			Within run	Within lab
Acinetobacter baumannii	Bacterium	791.12	20.94	26.34
Escherichia coli	Bacterium	17.76	17.39	19.09
Haemophilus influenzae	Bacterium	143.12	24.64	21.18
Klebsiella pneumoniae	Bacterium	17.76	17.43	22.62
Pseudomonas aeruginosa	Bacterium	127.59	18.97	20.22
Staphylococcus aureus	Bacterium	17.76	27.71	32.93
Streptococcus pneumoniae	Bacterium	631.29	21.83	16.36
Aspergillus fumigatus	Fungus	327.60	28.43	38.17
Candida albicans	Fungus	2.43	38.16	24.59
Human adenovirus	DNA virus	1,191.18	29.25	24.46
Mean	NA	NA	24.48	24.60

a The qualitative reproducibility of our assay was 100%. CV, coefficient of variation; NA, not applicable.

We demonstrated the within-run (repeatability) precision by testing four independent 1:125-diluted mock microbial community samples on the same run and the within-laboratory (reproducibility) precision by testing four repetitions of 1:125-diluted mock microbial community samples across four consecutive sequencing runs (Table 1 and Table S4). In terms of qualitative analysis, all 10 spiked organisms were detected in both the within-run and within-laboratory tests. Furthermore, we attempted to quantitatively analyze the coefficient of variation (CV) of the RPTM value for all 10 microorganisms. The average CV values for the 10 microorganisms were 24.48% (95% CI, 19.79% to 29.16%) within a run and 24.60% (95% CI, 19.89% to 29.30%) within the laboratory (Table 2).

The number of human cells varies greatly between BALF samples, which influences the analytical sensitivity of mNGS (12). Thus, we spiked 10^4^, 10^5^, and 10^6^ PBMCs/mL into 1:25-diluted mock microbial community samples to investigate the interference from the host nucleic acid (Table 1 and Table S4). All 10 organisms were successfully identified in each group. We further analyzed the change in internal DNA control (named UMSI) reads: when the number of host cells increased by 1 log, an approximately 1-log reduction appeared in the number of UMSI reads (Fig. S5a).

It is challenging to accurately distinguish genetically similar and clinically relevant microorganisms when coinfection occurs. Thus, a pair of related species in the same genus (Staphylococcus aureus and Staphylococcus epidermidis) was mixed at concentration ratios of 8:1, 1:1, and 1:8. Three repetitions for each ratio were tested, and the calculated RPTM ratios (RPTM_S. aureus_/RPTM_S. epidermidis_) were averaged (Fig. S5b). The mean RTPM ratios were 7.64 (95% CI, 6.23 to 9.04), 1.11 (95% CI, 1.04 to 1.19), and 0.20 (95% CI, 0.1593 to 0.240), and all ratios were within the range of a 2-fold change in the initial input ratio.

**FIG 3:**
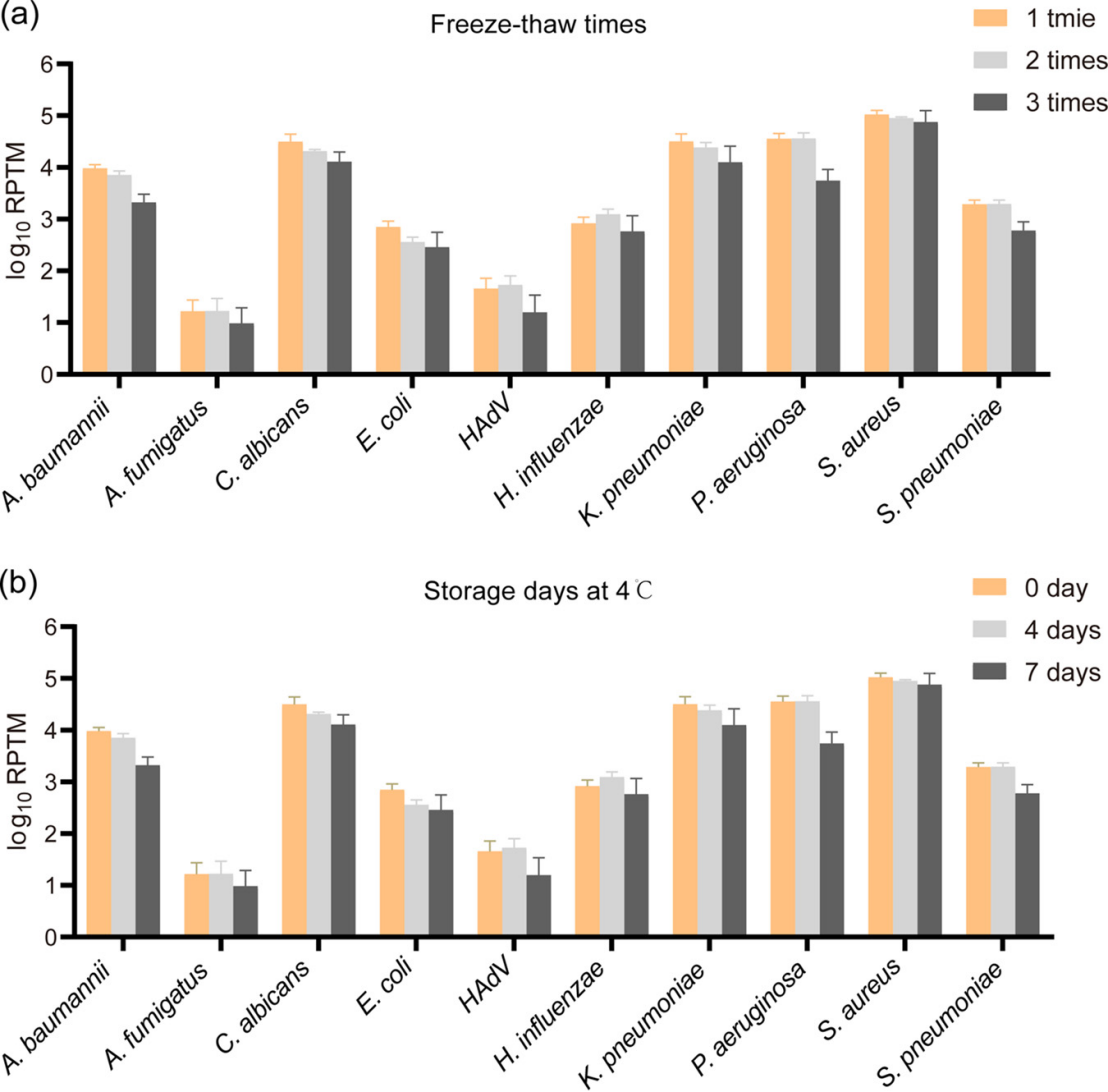
Stability analysis of mNGS. (a) Influence of freeze-thaw cycle times on pathogen detection. (b) Influence of storage time at 4°C on pathogen detection. *, *P* < 0.05. HAdV, human adenovirus; RPTM, number of mapped reads per 20 million.

We analyzed the replicates of the 1:25-diluted mock microbial community samples that were subjected to three freeze-thaw cycles and stored at 4°C for 0, 4, and 7 days (Table 1). All microbes were correctly identified in these samples. No difference was observed in the number of freeze-thaw cycles or RPTM values for all microbes (Fig. 3a). However, the RPTM gradually decreased when the storage time was increased at 4°C for most microbes (Fig. 3b). Significant differences were also observed in the RPTM values between samples stored at 4°C for 0 and 7 days for Candida albicans (*P = *0.039) and Acinetobacter baumannii (*P = *0.027).

**FIG 4:**
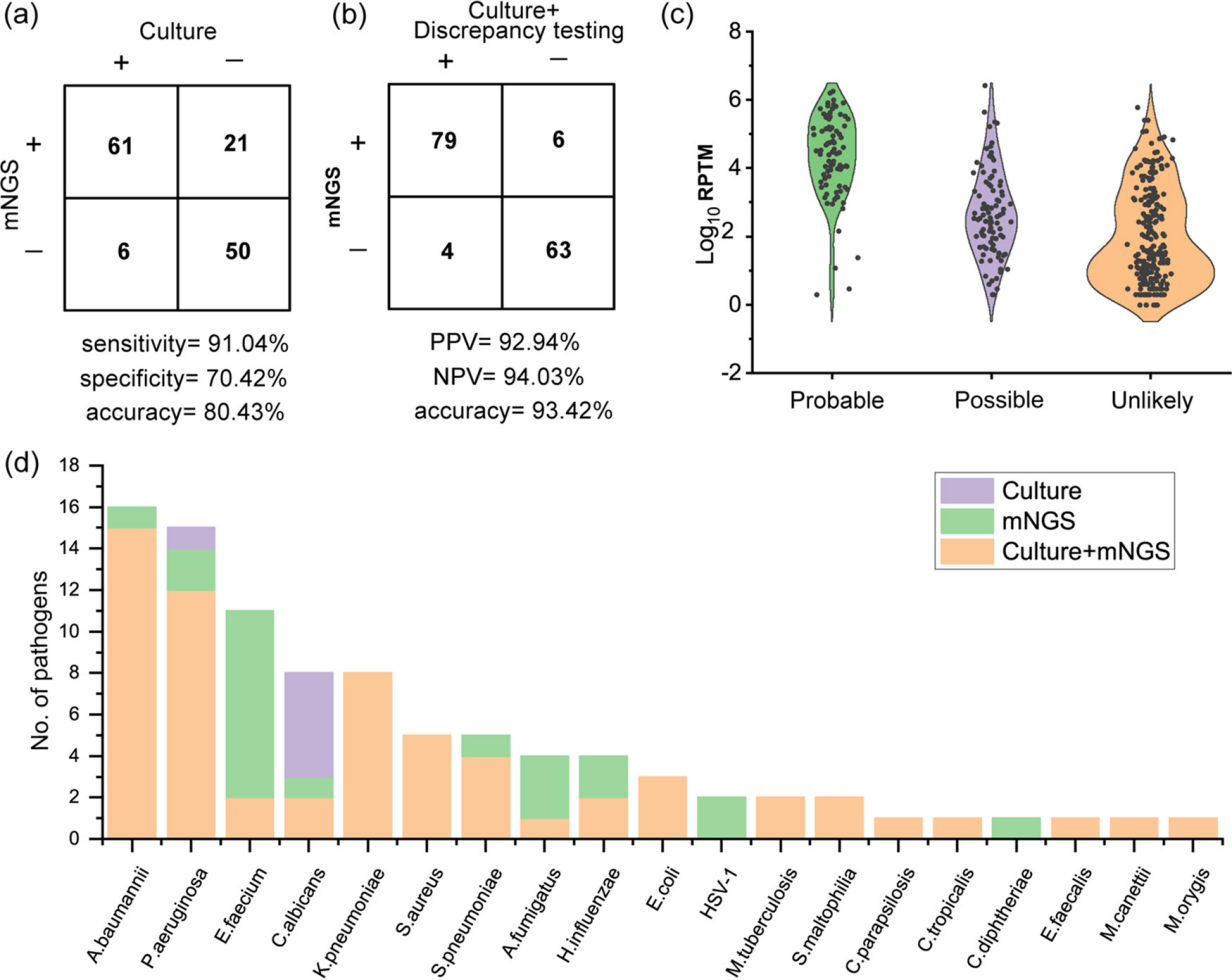
Accuracy analysis of mNGS. (a) Comparison of the performance of mNGS relative to clinical culture criteria. (b) Comparison of the performance of mNGS relative to a composite criterion. (c) Distribution of RPTM abundances of probable, possible, and unlikely pathogens. (d) Cumulative results of pathogens detected by culture and/or mNGS. HSV-1, herpes simplex virus 1; NPV, negative predictive value; PPV, positive predictive value.

To evaluate accuracy, a total of 43 culture-positive BALF samples and 26 culture-negative samples from 61 patients were tested by mNGS. Of the 61 patients, 39 were diagnosed as having LRIs and 22 were without LRIs (Table S5). The percent agreements of mNGS results with culture and composite standards were 80.43% and 93.42%, respectively. Considering clinical culture as the gold standard, mNGS had a sensitivity of 91.04% (95% CI, 80.88% to 96.31%) and a specificity of 70.42% (95% CI, 58.25% to 80.37%) (Fig. 4a). The conflicting results between culture and mNGS tests were subjected to quantitative PCR (qPCR) (Table S6). Compared with the composite reference criterion (culture plus qPCR), mNGS showed a sensitivity of 95.18% (95% CI, 87.45% to 98.44%), a specificity of 91.30% (95% CI, 81.40% to 96.42%), a positive predictive value of 92.94% (95% CI, 84.70% to 97.10%), and a negative predictive value of 94.03% (95% CI, 84.65% to 98.07%) (Fig. 4b). The RPTM for the probable pathogens was significantly higher than those of the possible and unlikely pathogens (Fig. 4c). Five C. albicans isolates and one P. aeruginosa isolate were cultured but were ignored by mNGS, among which four C. albicans isolates were determined to have false-negative results with cycle threshold (*C_T_*) values above 31 by follow-up qPCR. The other pathogens (C. albicans *and* P. aeruginosa) were reclassified as having true-negative results. Twenty-three culture-negative (or not available by culture, e.g., DNA virus) but mNGS-positive pathogens underwent discrepancy tests, most of which (73.91% [17/23]) were concordant with mNGS. Six pathogens (four Enterococcus faecium isolates, one Aspergillus fumigatus isolate, and one Corynebacterium diphtheriae isolate) were identified by qPCR and were determined to be false-positive results.

In this study, 67 pathogens were detected by microbial cultivation, of which bacteria and fungi accounted for 82.09% (55/67) and 17.91% (12/67), respectively. mNGS has a much wider pathogen detection spectrum than does microbial culture (Fig. 4d). Bronchoalveolar lavage fluid mNGS detected 86 probable pathogens from 47 samples, including 71 (82.55%) bacteria, 13 (15.11%) fungi, and 2 (2.32%) DNA viruses. The diagnostic performance of mNGS for bacterial detection was also estimated based on the composite reference standard, and the sensitivity and specificity were 100% (95% CI, 93.24% to 100%) and 92.75% (95% CI, 83.21% to 97.30%), respectively. Taking the composite reference criterion as the standard, mNGS has a sensitivity of 71.43% (95% CI, 42.00% to 90.41%) and specificity of 98.55% (95% CI, 91.11% to 99.92%) for fungal detection. In addition, two human alphaherpesvirus 1 molecules were detected by BALF mNGS with a high load (*C_T_* ≤ 20).

## DISCUSSION

In recent years, an increasing number of laboratory-developed mNGS methods have emerged and exhibited unique advantages in diagnosing infectious diseases (11, 13, 14). However, the standardization of mNGS for pathogen detection is progressing slowly. As no standard mNGS pipelines are approved, all existing laboratory-developed mNGS tests should be validated to demonstrate their performance characteristics. In this study, we designed an in-house DNA-based mNGS assay and validated it through the combination of metagenomic-specific and traditional validation strategies, including the use of hundreds of *in silico* simulations and published raw data sets from patients to test the accuracy of the bioinformatics pipeline and mock microbial communities and clinical samples to assess the analytical performance.

Well-defined reference materials are the cornerstone for performing quality control, whose main characteristics are uniformity and stability. The perfect biological reference materials for mNGS are residual patient specimens (5). In this study, a series of reference materials were developed to meet different evaluation purposes. Since a sufficient and uniform negative BALF matrix was unavailable, we utilized only a small number of spiked clinical BALF samples to evaluate the limited analytical performance (LoD and linearity). The similar performance evaluated by spiked clinical samples and mock microbial community samples indicated that utilizing the simulated microbial community samples could also reflect the mNGS performance to some extent when the clinical samples were inadequate.

The analytical sensitivity of clinical mNGS is influenced by several factors, such as the extraction efficiency, library preparation bias, genome size, and the availability of matching reference genomes in the database (7). This study presented the assay limits of detection of 10 common pathogens in LRIs ranging from 2.4 copies/mL for C. albicans to 1,191.2 copies/mL for HAdV. Gu et al. demonstrated that the LoD values for their mNGS assay were 400 to 700 genome equivalents (GE) mL^−1^ for bacteria and 4 GE mL^−1^ for fungi, which were consistent with our data (15). However, Miller et al. reported that the LoD for their mNGS assay was estimated to be approximately 10 CFU/mL for bacteria, which seems more sensitive than our mNGS assay (7). The amount of human nucleic acid between our mock BALF matrix and synthetic CSF matrix was different, which greatly influenced the analytical sensitivity. In addition, inconsistency in the measurement units of bacteria may also cause this difference because 1 CFU may mean dozens to hundreds of copies between various bacterial strains (16). In this study, C. albicans showed an extremely low LoD (2.42 copies/mL) compared with those of other organisms, which is consistent with the results of the study by Miller et al. (0.2 CFU/mL) (7). The high sensitivity of C. albicans was attributed to its larger genome and low threshold, as only a small amount of C. albicans exists in the laboratory environment. Similarly, A. fumigatus also had the above characteristics, but the hard cell wall hindered the release of bacterial DNA and resulted in a higher LoD. The mNGS assay exhibited lower sensitivity for the detection of DNA viruses. The introduction of chemical reagents to selectively lyse human cells may also destroy the integrity of the virus to some extent, and the released nucleic acids from humans and viruses are digested by Benzonase.

In this report, we presented the within-run and within-laboratory precision of our mNGS assay in both qualitative and quantitative analyses. In the qualitative analysis, all 10 microbes were detected in each batch, and the qualitative precision achieved 100%, which was consistent with the CV of the mNGS assay developed by Miller and colleagues for pathogen detection in CSF samples (7). Judging from the quantitative aspects, Blauwkamp et al. reported that the CV for their microbial cell-free DNA-based mNGS tests ranged from 16.7 to 18.9% (mean, 18.2%) within a run and 17.9 to 22.2% (mean, 19.9%) within the laboratory (8). The inferior precision of our mNGS assay compared to that of microbial cell-free DNA-based mNGS was understandable, as the parameters used to calculate the precision were different. Our study used the mapping reads per 20 million (RPTM) for each microbe to calculate the precision, while Blauwkamp et al. utilized the molecules of microbe-specific cell-free DNA (cfDNA) per microliter of plasma (MPM). Thus, the precision of the two studies lacked comparability. In addition, the process of extracting nucleic acids from intact pathogens is a known source of variation between microbial community characterization experiments (17). According to the FDA, the precision around the mean value should not exceed 15% of the CV for quantitative experiments (18). Therefore, it was more rational to consider the mNGS assay as a qualitative experiment according to our results.

Compared with traditional pathogen culture, mNGS had a significantly higher sensitivity (91.04% [95% CI, 80.88% to 96.31%]) but a lower specificity (70.42% [95% CI, 58.25% to 80.37%]). However, when considering culture plus qPCR as the gold standard, the sensitivity and specificity of mNGS increased to 95.18% (95% CI, 87.45% to 98.44%) and 91.30% (95% CI, 81.40% to 96.42%), respectively. Wang et al. enrolled 55 cases to compare the performance of mNGS and conventional tests for mixed-pneumonia diagnosis (19). They found that mNGS had much higher sensitivity (97.2% versus 13.9%; *P < *0.01) and a lower specificity (63.2% versus 94.7%; *P = *0.07) than the conventional test. The lower specificity of mNGS compared with culture may be because traditional pathogen culture has low clinical sensitivity (50% to 80%). Most “false-positive results” of mNGS (71.43% [15/21]) were proven to be true positives by qPCR in our study.

Because mNGS is an unbiased detection method, it is inevitable to be puzzled by the manner of distinguishing the colonization, contamination, or etiological agents, which influences the analytical specificity of mNGS assays. Strict physical separation and extensive cleaning of laboratory surfaces with 10% sodium hypochlorite are necessary. In this study, a no-template control and negative control were incorporated into every individual sequencing run to monitor the source of contamination. Thus, a unique background microorganism list was generated, which contributed to the interpretation of further studies. One of the challenges for many taxonomic classifiers is reporting large numbers of low-abundance false positives, which lowers the precision of mNGS (20). Generally, a robust threshold can reduce these interferences while sacrificing analytical sensitivity. We used ROC curve analysis to establish the optimal threshold, thus allowing the differentiation of pathogens from the background noise and maximizing the sensitivity. Moreover, physicians and microbiologists need to be familiar with common pathogens, conditional pathogens, and normal microbes in the respiratory tract. A good judgment is the result of a comprehensive evaluation, such as the number of reads and genome coverage, clinical symptoms, and patient immunity status (3).

Preanalytical procedures, including BALF collection, transport, and storage, are crucial for pathogen detection. Long-term storage at 4°C and repeated freezing-thawing showed a tendency to reduce the RPTM, which may lead to false-negative results, especially for low-microbial-biomass samples. The American Thoracic Society provided recommendations for the performance and processing of BALF, which indicated that the specimens should be transported and stored at 4°C for up to 24 h (21).

The interference from overwhelming human nucleic acids and the high degree of genetic similarity between species were also assessed. Elevated levels of human-derived cells in samples influence the analytical sensitivity and increase the sequencing cost (22). The sharp reduction in UMSI reads indicated that a high host background was present and that negative mNGS results may be less useful for excluding infection; other confirmed tests that are less influenced by background are recommended (7). Genetically related microbes may cause cross-reactivity for pathogen identification, which influences the analytical specificity. The presence of background microorganisms, bias from nucleic acid extraction and library preparation, and misclassification in bioinformatics analysis would result in a discrepancy between the acquired ratios (RPTM_S. aureus_/RPTM_S. epidermidis_) with the input ratios (8).

To our knowledge, this study is the first to comprehensively validate the mNGS assay for pathogen detection from BALF samples. A large number of clinical laboratories have developed in-house mNGS assays for the diagnosis of infectious disease and have shown excellent diagnostic performance for infectious disease (10, 13, 14, 19, 23). However, most clinical mNGS studies only focused on the evaluation of diagnostic performance and overlooked the analytical validation of their mNGS methods to demonstrate their analytical performance. This study established an in-house mNGS method for the diagnosis of LRIs from BALF samples and exhaustively described how to prepare *in silico* and biological reference materials and how to develop comprehensive validation schemes for in-house mNGS assays. Although the mNGS test was still a qualitative test, we attempted to present both qualitative and quantitative analyses to evaluate the analytical performance of the mNGS assay, which is helpful to explore the relationship between the pathogen loads and the mNGS signals. We involved 10 representative pathogens in lower respiratory tract infection and established the analytical performance for each pathogen. In addition, an efficient and simple test plan was developed for analytical validation in which one mock BALF sample reflected various analytical performances. Our study provided valuable experience for validating laboratory-developed mNGS assays, which will foster the development of better practices and contribute to the standardization of mNGS methods. This study also has some limitations. We only validated an mNGS assay to identify the pathogens in BALF samples in this study, as the workflow was easier and the requirements were more urgent than for RNA-based mNGS. Viruses are also an important cause of LRIs, and using PCR to detect viral respiratory infections is a current routine practice. In addition, the relatively small size for clinical samples may lead to a reporting bias.

### Conclusions.

In summary, this study presented a ready-made example for clinical laboratories to prepare reference materials and develop validation schemes for their LDTs in the diagnosis of LRIs. Well-defined reference materials are the premise for performing validation, and a comprehensive validation scheme can promote the optimization of methodology. A fully validated and well-behaved mNGS workflow will aid in the diagnosis of infectious diseases and improve patient management. Performing validation will also facilitate the development of more perfect mNGS pipelines for clinical care. Large-scale prospective studies are required to demonstrate the clinical utility of this emerging technology. With the increasing standardization and development of technology, the application of mNGS has shifted from laboratories to clinical settings, which will contribute to precision diagnosis and tailored antibiotic therapy for clinical infectious diseases.

## MATERIALS AND METHODS

### mNGS assay.

A standard operating procedure of the DNA-based mNGS method was developed for the diagnosis of LRIs. Briefly, 1 mL of sample was centrifuged at 12,000 × *g* for 5 min to collect the pathogens and human cells. Next, 50 μL of precipitate underwent depletion of host nucleic acid using 1 U of Benzonase (Sigma) and 0.5% Tween 20 (Sigma) and incubated at 37°C for 5 min (1). Terminal buffer (400 μL) was added to stop the reaction. Then, the quantified unique DNA fragments (named UMSI) were spiked for each sample as an identity and internal control, which were PCR products of Oryza sativa 400 to 600 bp in length. A total of 600 μL of the mixture was transferred to new tubes containing 500 μL of ceramic beads for bead beating using a Minilys personal TGrinder H24 homogenizer (catalog number OSE-TH-01; Tiangen, China). Then, the nucleic acid from 400 μL of pretreated samples was extracted and eluted in 60 μL of elution buffer using a QIAamp UCP pathogen minikit (catalog number 50214; Qiagen, Germany). The extracted DNA was quantified using a Qubit double-stranded DNA (dsDNA) high-sensitivity (HS) assay kit (catalog number Q32854; Invitrogen, USA).

Thirty microliters of the eluate was used to generate libraries using the Nextera DNA Flex kit (Illumina, San Diego, CA, USA) according to the manufacturer’s instructions. Fragmentation and tagmentation of the DNA were performed using the bead-linked transposome. After completion of posttagmentation cleanup, the tagmented DNA was amplified; the thermocycling parameters were as follows: 68°C for 3 min and 98°C for 3 min, followed by 18 cycles of 45 s at 98°C, 30 s at 62°C, and 2 min at 68°C, before a final minute at 68°C. Dual indexing was conducted by employing the IDT for Illumina DNA/RNA UD indexes (catalog number 20027213). Purification and size selection were carried out following the double-sided bead purification procedure. A Qubit dsDNA HS assay kit was used to measure the library concentration. Library quality was assessed with an Agilent 2100 Bioanalyzer (Agilent Technologies, Santa Clara, CA, USA) using a high-sensitivity DNA kit. The library was prepared by pooling a 1.5 pM concentration of each purified sample equally for sequencing on an Illumina NextSeq 550 sequencer using a 75-cycle single-end sequencing strategy.

For bioinformatics analysis, Trimmomatic was used to remove low-quality reads, adapter contamination, duplicate reads, and reads shorter than 70 bp (24). Low-complexity reads were removed by Kcomplexity using default parameters. The human sequence data were identified and excluded by mapping to a human reference genome (hg38) using SNAP v1.0beta.18 (25). To construct the microbial genome database, pathogens and their genomes or assemblies were selected following the Kraken2 criteria for selecting representative assemblies for microorganisms (bacteria, viruses, fungi, protozoa, and other multicellular eukaryotic pathogens) from the NCBI Assembly and Genome databases (https://benlangmead.github.io/aws-indexes/k2). All accession numbers of our database are provided in Table S7. Microbial reads were aligned to the database using Burrows-Wheeler Aligner software (26). We defined that reads with 90% identity of reference were mapped reads. In addition, reads with multiple locus alignments within the same genus were excluded from the secondary analysis. Only reads mapped to the genome within the same species were considered.

We normalized the sequencing reads RPTM to eliminate the errors caused by various sequencing depths among samples. To establish the optimal threshold value for the >10 microbes with culture isolates, samples spiked with microbes were defined as positive samples, while negative control (NC) was defined as the negative sample. Receiver operating characteristic curves were plotted for each target species using these samples. The parameter resulting in the highest area AUC was considered the positive cutoff value for this species (27). For microorganisms without culture isolates, the RPTM mean value and standard deviation of this microorganism were calculated, and the RPTM (mean + 2 standard deviations [SD]) was set as a positive cutoff value (10).

The clinical reportable range (CRR) for pathogens was established according to the following three references indicated in a previous study (10): (i) the Johns Hopkins ABX Guide (https://www.hopkinsguides.com/hopkins/index/Johns_Hopkins_ABX_Guide/Pathogens), (ii) *Manual of Clinical Microbiology* (28), and (iii) clinical case reports or research articles published in peer-reviewed journals. All microbes that exceeded the threshold of mNGS were classified into 3 categories (13): (i) probable (BALF mNGS-based results were within the CRR and concordant with the clinical and radiologic results; the RPTM was significantly higher than the positive cutoff value, and the abundance was obviously higher than that of other species of the same genus), (ii) possible (the microbe has pathogenic potential, but an alternate explanation is more likely), and (iii) unlikely (the microbe cannot cause pneumonia).

To monitor the sources of potential contamination, both NC and sterile deionized water, which served as nontemplate controls, were prepared in parallel with other samples in each batch (7). In addition, we used sterile cotton swabs dipped in sterile deionized water to wipe the surfaces of the centrifuge and biosafety cabinet to generate the background microorganism list in our laboratory.

### Metagenomic bioinformatics pipeline validation.

We prepared three data sets for dry-laboratory validation at the single-genome, multiple-genome, and clinical-metagenome levels. In data set I, we first picked 309 species that are well known to infect human beings (see Table S3 in the supplemental material). The bioinformatics simulation method was as follows. (i) Pathogens included 171 bacteria, 78 viruses, 33 fungi, and 27 parasites. (ii) The species were required to contain a complete genome and were downloaded from the NCBI GenBank database. (iii) Bacteria, fungi, and parasites were randomly picked up 1,000,000 reads, and viruses were generated from 1,000 reads.

In data set II, we simulated 100 samples and generated the following criteria: (i) each sample contained 20 pathogens randomly selected from 309 reference pathogens, (ii) the read number of each pathogen was randomly chosen, (iii) reads were randomly picked up from the reference genome according to the number generated at step 2, (iv) 20 million host reads were also generated and added to each sample, and (v) all random functions were based on Poisson distribution. In data set III, we involved a public pneumonia cohort (11).

Simulated reads for the misclassification test were created using wgsim v1.8 with default parameters for a total of 1 million single-end reads and 1 thousand reads from each assembly of the microbial genome. The Kraken2 database was downloaded from https://benlangmead.github.io/aws-indexes/k2 and included archaea, bacteria, viruses, plasmids, humans, protozoa, fungi, and plants. The Kraken2 command was as follows: kraken2 –threads 32 –fastq-input –confidence 0.05 –bzip2-compressed input_1.fastq input_2.fastq –output output.reads –report output.report. The Bracken command was as follows: est_abundance.py -i input -k db -o output.krakenreport.

The most important metrics for metagenomic classification are precision and recall. These metrics are chosen because they focus on the positive class of identified taxa, and typically not much can be said about the negative class that contains unknowns. Precision is defined as the proportion of true classified taxa over all classified taxa. Recall is the proportion of correctly classified abundances over all true abundances. The F1 score was calculated by the following formula:

$$\text{F}1\text{score}=\frac{2\times \text{precision}\times \text{recall}}{\text{precision}+\text{recall}}$$

### Preparation of biological samples.

This study utilized three types of biological samples to evaluate an in-house mNGS method for pathogen detection from BALF samples. First, a few negative clinical BALF materials were spiked with three known concentrations of microbes to prepare the spiked samples. However, it is impossible to obtain a large number of homogeneous clinical BALF samples as a sample matrix. Thus, we designed a series of mock microbial community samples to comprehensively validate the basic analytical performance of mNGS. In addition, 69 clinical BALF samples with clinical laboratory testing results were used to assess the accuracy of mNGS. The preparation details of various samples were as follows.

Ten representative microbes with well-defined sources in LRIs were enrolled in samples; these microbes showed a broad range of GC content ranging from 30% to 70% and had genomes spanning from kilobases to megabases in length (Table S8). All bacteria and fungi were cultured under standard conditions, as outlined previously (29). The colonies were scraped from the culture plate with disposable buccal swabs and added to sterile saline solution (0.9% [wt/vol] NaCl) to prepare the original suspensions. A pure spore suspension of A. fumigatus and a culture supernatant of human adenovirus were directly used for nucleic acid extraction and quantification. The EvaGreen dye-based Bio-Rad QX200 droplet digital PCR (ddPCR) system (Bio-Rad, USA) was used to quantify the concentration of the 10 microbial suspensions. The primers for the above microbes are available in Table S9.

For spiked clinical BALF samples, two S. pneumoniae and P. aeruginosa negative BALF samples were pooled, and human nucleic acids were quantified by ddPCR. We diluted the pooled clinical sample matrix with sterile normal saline to ensure that the amount of human nucleic acid was equal to that in the mock microbial community samples (10^5^ cells/mL). A 5-fold dilution series of S. pneumoniae and P. aeruginosa was added to the clinical BALF mixture (Table S4).

A series of mock microbial community samples were designed to perform the analytical validation (Table S4). Each of the 10 representative pathogens was mixed and diluted with sterile saline solution (0.9% [wt/vol] NaCl) 5-fold. To mimic the BALF samples, PBMCs were added to each community according to the experimental design at a concentration from 1 × 10^4^ to 1 × 10^6^ cells/mL. The PBMCs were prepared by using red blood cell lysis buffer (catalog number R1010; Solarbio, China) to lyse the red blood cells in whole human blood. The cell pellets were washed and resuspended in sterile saline solution (0.9% [wt/vol] NaCl), and the concentration was determined using a hemocytometer. To reduce the cost and simplify the validation workflows, a series of samples were diluted, and one dilution was used to test various performance characteristics (Table 2).

Sixty-nine residual BALF samples from 61 patients with clinical laboratory testing results were collected from January to August 2021. The BALF samples were collected in sterile wide-mouth containers with volumes greater than 2 mL. Based on the outcomes of microbiological culture, 43 positive and 26 negative BALF samples from 61 patients with suspected LRIs were included. This study was approved by the Medical Ethics Review Committee of Beijing Hospital (2021BJYYEC-128-01).

### Validation of the whole mNGS assay.

This study utilized spiked clinical BALF samples and mock microbial communities to estimate the LoD and linearity of mNGS, respectively. For spiked clinical BALF samples, a 5-fold dilution series of S. pneumoniae and P. aeruginosa (from 10,000 to 32 copies/mL) was spiked into a mixture of two pooled negative BALF samples (Table S4). For mock microbial communities, the microbial suspensions with high concentrations were diluted by mock-negative BALF matrix in a series of 5-fold dilutions from 1:1 to 1:78,125 (Table S4) (15). Each concentration of microorganism was tested in four replicates. The LoD was calculated as the concentration at which mNGS detected pathogens in 95% of replicates.

The assay linearity was also evaluated by performing a linear regression analysis on the same four sets of serially diluted samples used in LoD calculation. The log_10_-transformed total input organism mass (copies per milliliter) was plotted against the log_10_-transformed sequencing reads (RPTM). The best-fit regression line was plotted using GraphPad Prism 9, and the linear equation and *R*^2^ values were calculated using IBM SPSS Statistics 24.

The concentration of 1:625-diluted samples in this study may be near the LoD of some microbes (7). Thus, samples with concentrations higher than 1:125 were chosen to evaluate precision, which was tested in four replicates within a single run (repeatability) and across four different runs (reproducibility).

To assess the interference of human nucleic acid background in mNGS assay performance, low (10^4^ cells/mL), medium (10^5^ cells/mL), and high (10^6^ cells/mL) titers of PBMCs were spiked into the 1:25-diluted samples. In addition, the extent of cross-reactivity between genetically similar species during coinfection was also evaluated. The coinfection of S. epidermidis with S. aureus was mimicked by mixing samples at concentration ratios of 8:1, 1:1, and 1:8. The RPTM_S. aureus_/RPTM_S. epidermidis_ ratios were compared with the expected input ratios.

To evaluate the stability of the assay, three replicates of the 1:25-diluted samples were subjected to three freeze-thaw cycles and stored at 4°C for 0, 4, and 7 days. The acquired RPTM for each microbe was compared among the virus treatment conditions.

The assay accuracy was evaluated using 69 clinical BALF samples, comprising 43 positive samples and 26 negative samples. The mNGS assay detected probable pathogens that were compared with the results of conventional BALF culture, and any discordant results were evaluated using pathogen-specific quantitative PCR assays, which were considered correct. In this study, two reference criteria were established for comparison (7): (i) a clinical gold standard based on microbiological culture results and (ii) a composite criterion that combines the results from clinical culture and additional discrepancy tests. A specific scoring algorithm was adopted to evaluate the accuracy of mNGS as reported in a previous study (Table S10) (15): compared with the clinical gold standard or composite criterion, true positives or false negatives were counted for each microbe detected or not detected by mNGS, respectively. A true negative was scored if no other microbes were detected by mNGS, except for the expected pathogen(s). One or more false-positive results in a sample were scored as one false positive.

### Statistical analysis.

In this study, the Wilcoxon test and Kruskal-Wallis test were used to compare the differences across subgroups. IBM SPSS Statistics 24 was used to analyze the data, and a *P* value of <0.05 was considered significant.

### Data availability.

Raw data of mock and pathogen reads of clinical samples were deposited in the Genome Warehouse in the National Genomics Data Center (National Genomics Data Center Members and Partners, 2021) under project PRJCA007286 and are publicly accessible at https://ngdc.cncb.ac.cn/bioproject/browse/PRJCA007286.
