# Supplementary material for: Validation of a Metagenomic Next-Generation Sequencing Assay for Lower Respiratory Pathogen Detection
Source: Microbiol Spectr. 2022 Dec 12;11(1):e03812-22. doi: 10.1128/spectrum.03812-22 (PMC9927246; doi:10.1128/spectrum.03812-22)
Supplement: Supplemental file 1 — Supplemental material. Download spectrum.03812-22-s0001.pdf, PDF file, 1.5 MB [file spectrum.03812-22-s0001.pdf]

Supplementary Material for "Validation of a metagenomic next-generation sequencing assay for lower respiratory pathogen detection "

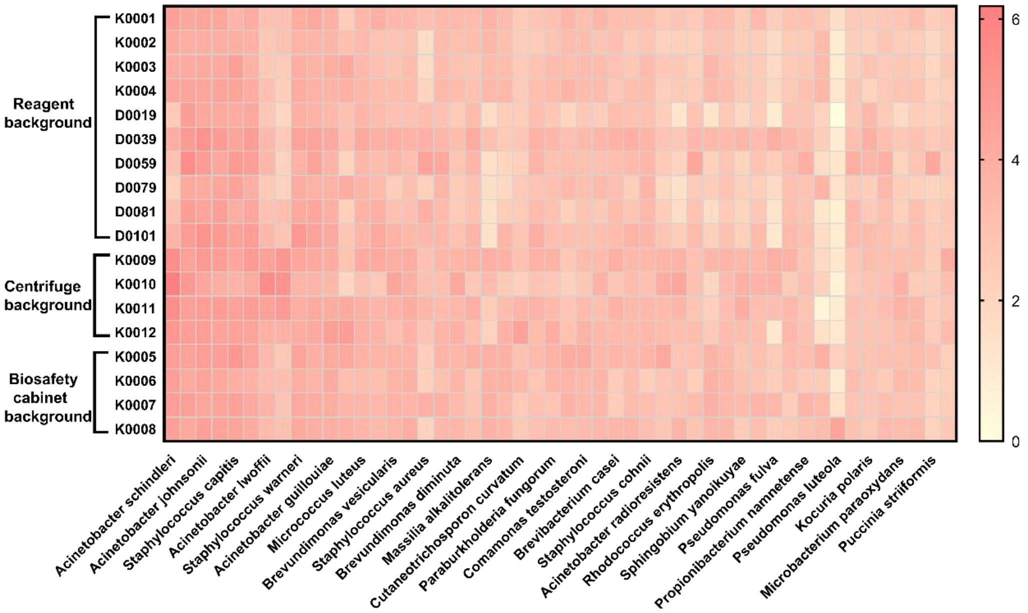

Fig. S1 Heatmap of the top 25 background microorganisms in our laboratory.

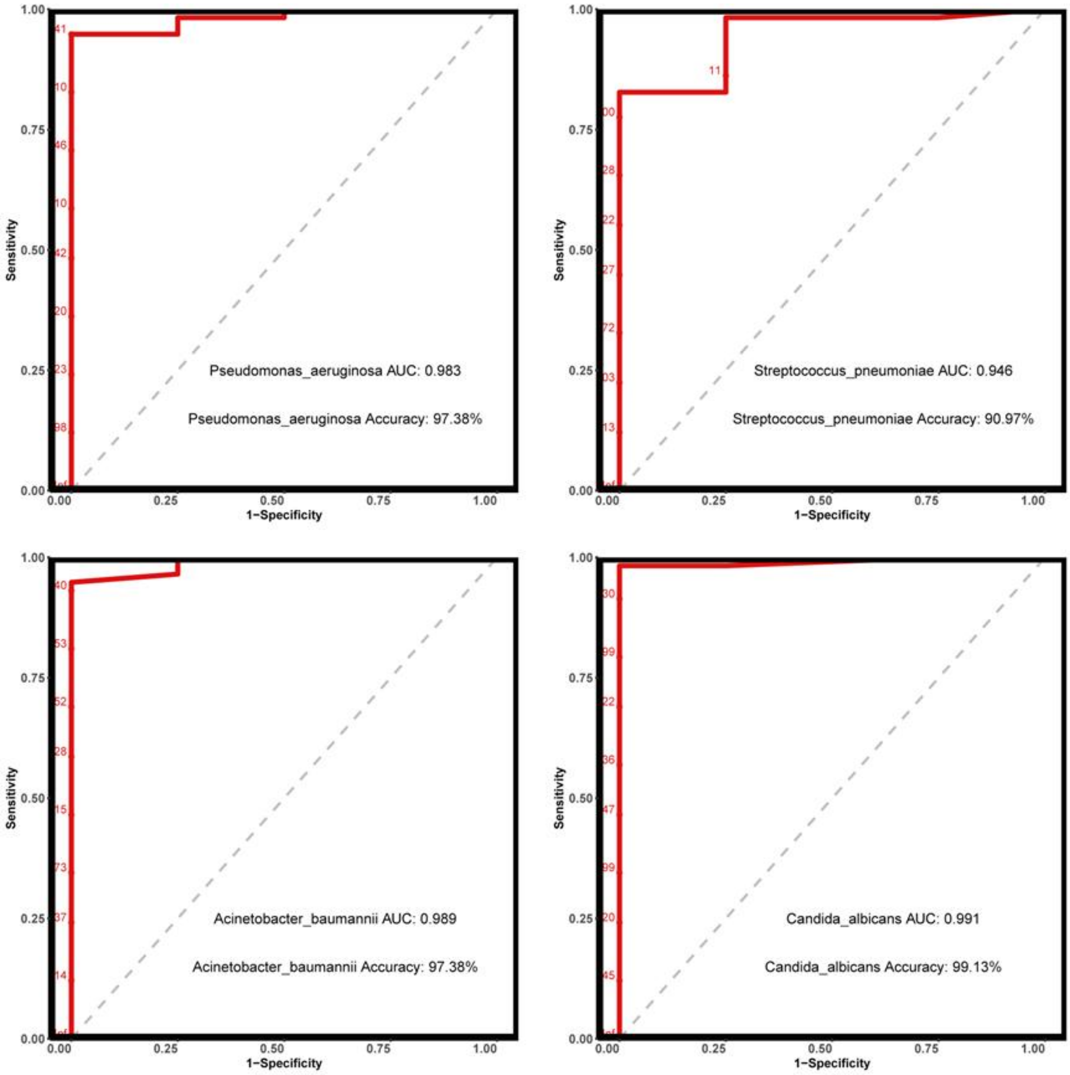

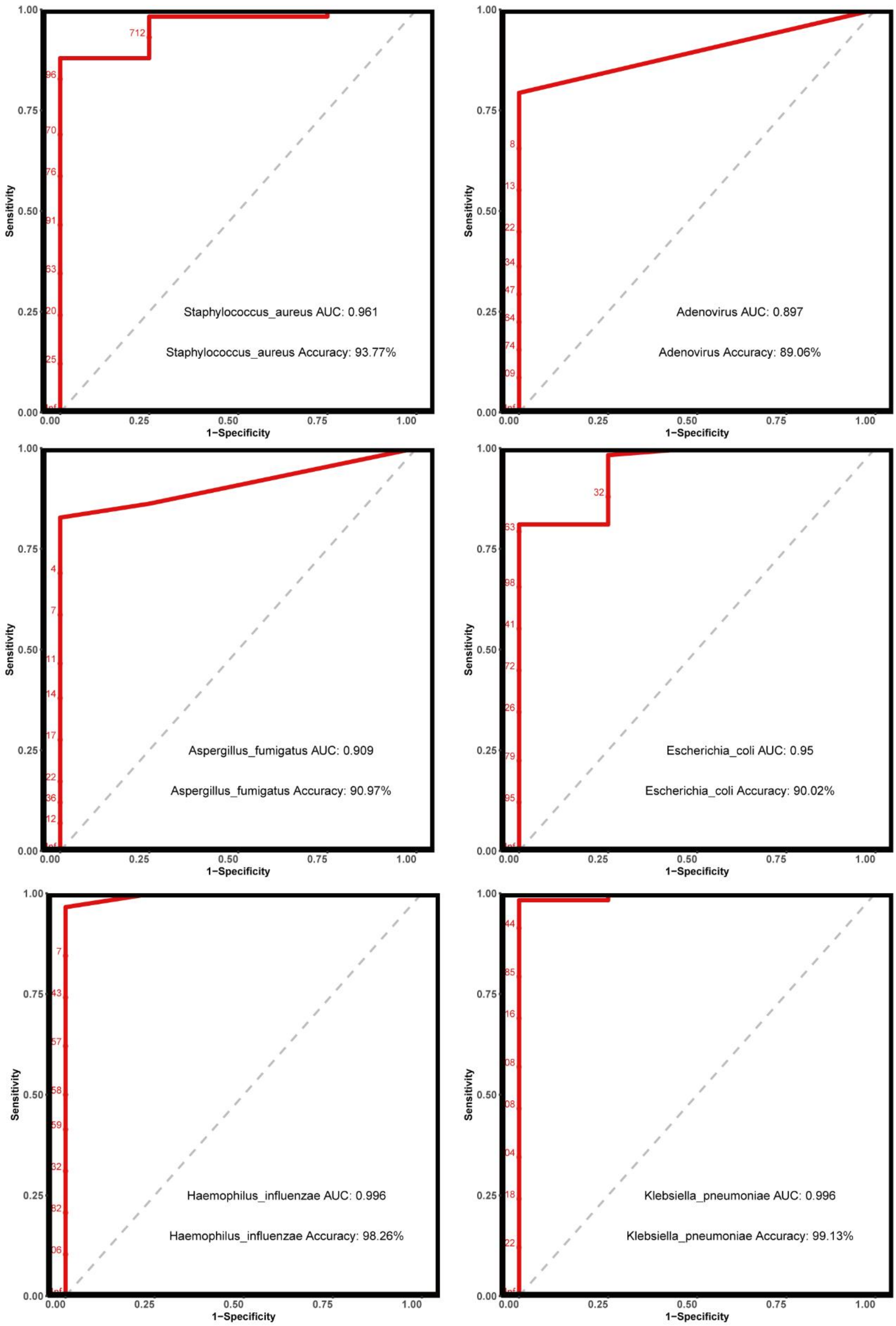

Fig. S2 The ROC curves for the spiked ten microbes.

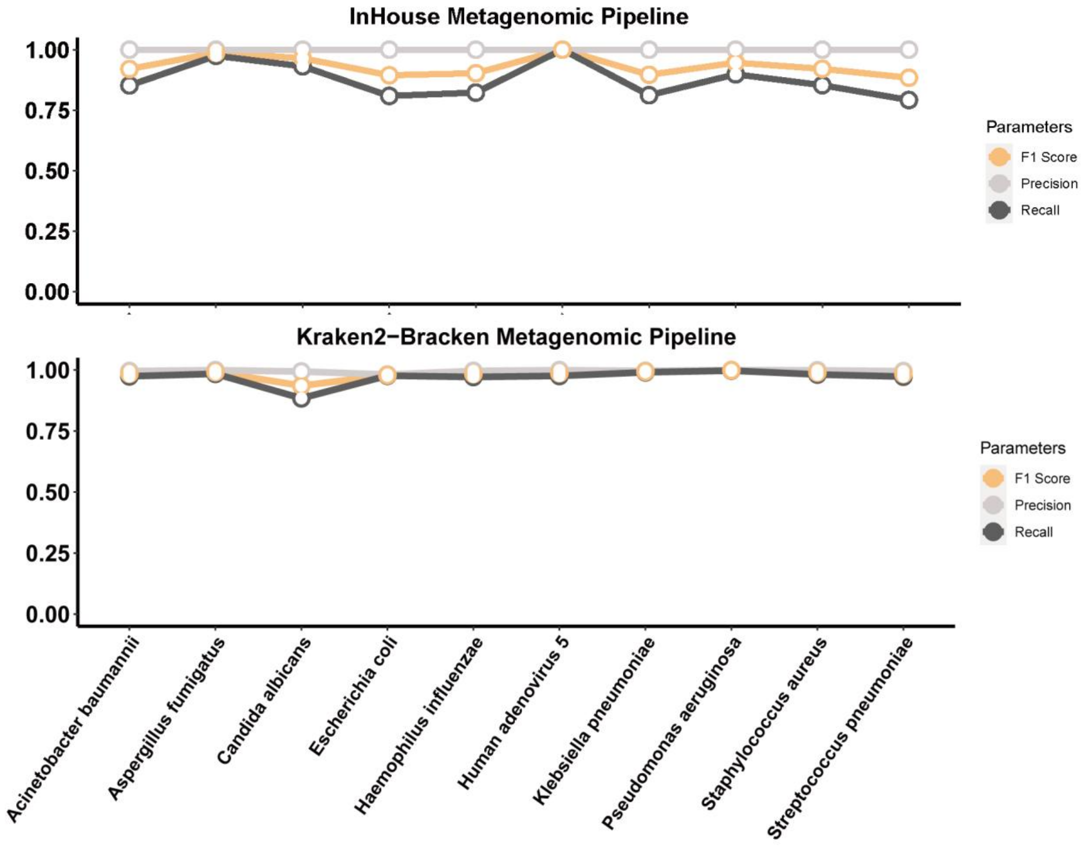

Fig.S3 The performance of in-house metagenomics pipeline and Kraken2-Bracken pipeline evaluated by single-genome stimulated data.

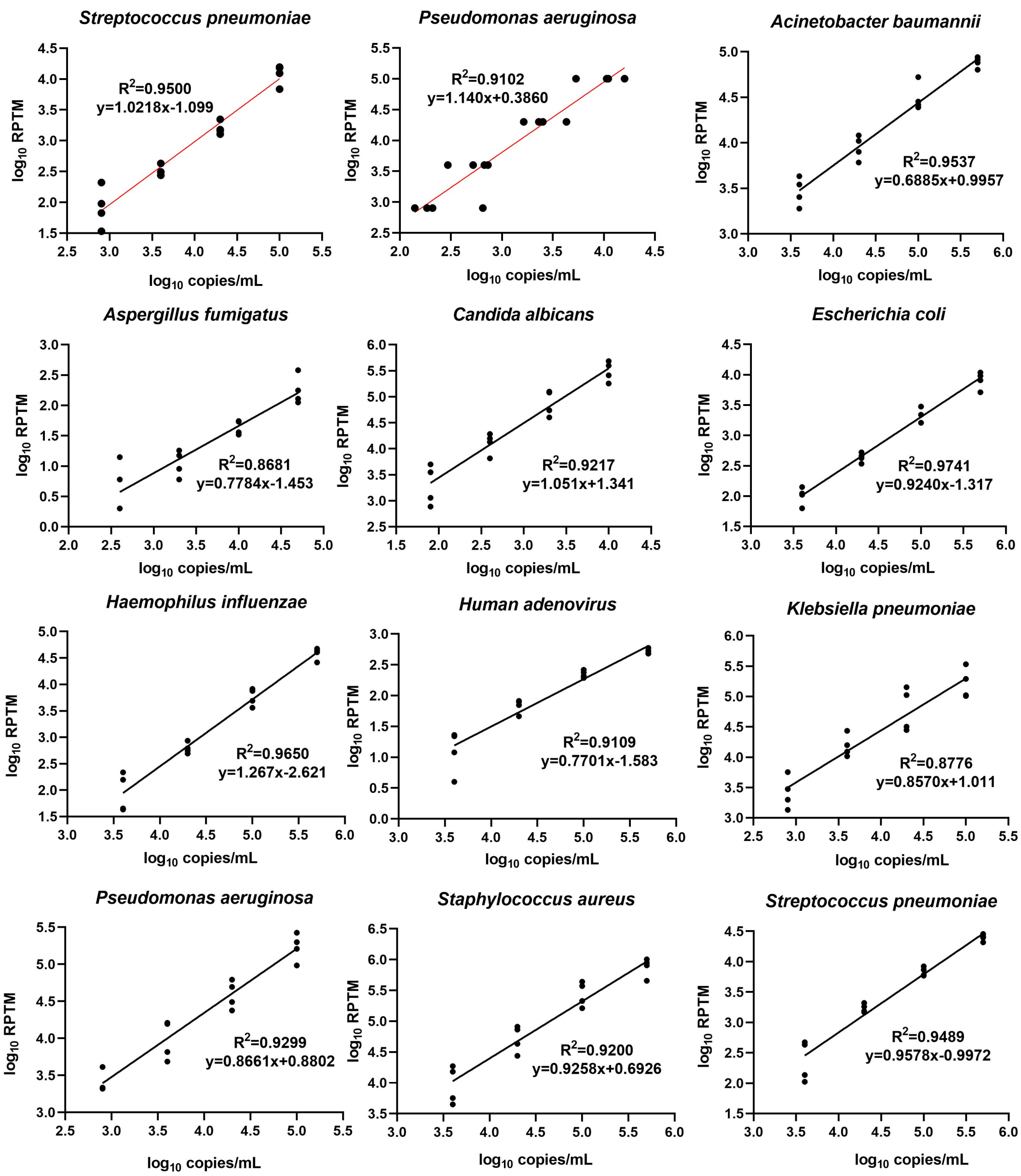

Fig S4. Relationship of the microbes titer with mNGS detection signal (expressed in RPTM). The red line was evaluated by spiked clinical BALF samples and the others were assessed by mock microbial community samples. RPTM, the numbers of mapped reads per twenty million.

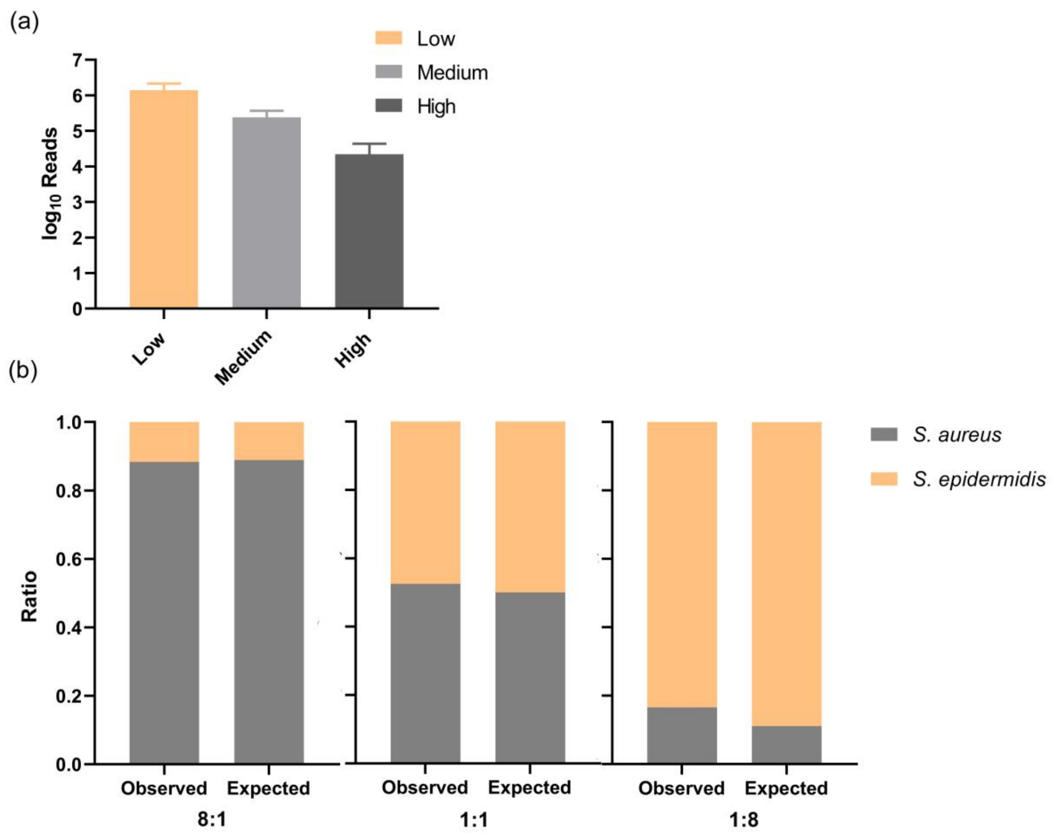

Fig S5. (a) Relationship of the acquired UMSI reads with the number of human cells. (b) The performance of mNGS in distinguishing closely related species.

Table S1. The list of background microorganisms.This table can be found in the separate Supplementary tables Excel file, in Tab 1

Table S2. Thresholds for the 10 represent microbes.

Species Name	Max Accuracy (%)	AUC	Thresholds (RPTM)
Acinetobacter baumannii	90.972	0.905	115
Aspergillus fumigatus	92.848	0.931	0.5
Candida albicans	99.134	0.996	4.5
Escherichia coli	91.780	0.972	14.5
Haemophilus influenzae	98.261	0.995	2.5
Human adenovirus	89.056	0.897	1
Klebsiella pneumoniae	99.134	0.998	16.5
Pseudomonas aeruginosa	97.379	0.980	139
Staphylococcus aureus	99.134	0.993	296
Streptococcus pneumoniae	90.972	0.945	26

AUC, Area under curve; RPTM, the number of mapped reads per twenty million.

Table S3. Bioinformatics validation of Kraken2 and in-house pipeline.This table can be found in the separate Supplementary tables Excel file, in Tab 2.

Table S4. The composition of spiked clinical BALF samples and mock microbial community samples for analytical validation.This table can be found in the separate Supplementary tables Excel file, in Tab 3.

Table S5. Clinical characteristics of the 61 patients.

	LRTIs（n=39）	Non-LRTIs (n=22)	P
Age, mean (range), years	66.7 (30-97)	62.0 (39-74)	0.220
Sex, male, n (%)	23 (60.0)	12（54.5）	0.737
Sex, female, n (%)	16（41.0）	10（45.5）	
Patients with identified pathogen			
Any pathogen	0	22（100%）	
Bacteria only	31（50.8%）	0	
Fungi only	3（4.9%）	0	
Multiple pathogens	5（8.2%）	0	
Hospital, mean (range), days	23.4（1-87）	10.5（2-30）	0.021
WBC, *10^9/L			0.084
<4	5（12.8%）	5(22.7%)	
4-10	25（64.1%）	13(59.1%)	
>10	9（23.1%）	4(18.2%)	
Neutrophil, %			0.501
40%-75%	21 (53.8%)	14(63.6%)	
>75%	18 (46.2%)	8(36.4%)	
Lymphocyte, %			0.013
<20%	35 (89.7%)	11(50.0%)	
20%-50%	4 (10.3%)	11(50.0%)	
CRP* , mg/L	43.1 (0.6-200)	16.4(0.1-109)	0.032
<10 mg/L	4 (11.8%)	7(43.8%)	
10–50 mg/L	19 (55.9%)	8(50.0%)	
51–100 mg/L	7 (20.6%)	0	
>100 mg/L	4 (11.8%)	1(6.3%)	
Procalcitonin*, ng/mL	5.4 (Undetected-100)	0.29 (Undetected-3.04)	0.005
<0.1 ng/mL	10 (32.3%)	10 (76.9%)	
0.1–0.24 ng/mL	4 (12.9%)	1 (7.7%)	
0.25–0.5 ng/mL	7 (22.6%)	1 (7.7%)	
>0.5 ng/mL	(32.3%)	1 (7.7%)	

*Some patients had no data. CRP , C-reactive protein; LRTI, lower respiratory tract infection.

Table S6. Pathogens identified by various methods.This table can be found in the separate Supplementary tables Excel file, in Tab 4.

Table S7. The list of accension ID of our database.This table can be found in the separate Supplementary tables Excel file, in Tab 5.

Table S8. The details of 10 microbes in the mock BALF samples.

Microorganisms	Class	%GC	Genome size	Source
Staphylococcus aureus	Gram Pos Bacteria	32.7	2.84Mb	ATCC 4330
Streptococcus pneumoniae	Gram Pos Bacteria	39.6	2.09 Mb	ATCC 49619
Staphylococcus epidermidis	Gram Pos Bacteria	32	2.51Mb	Clinical strain
Acinetobacter baumannii	Gram Neg Bacteria	39.0	3.97 Mb	Clinical strain
Pseudomonas aeruginosa	Gram Neg Bacteria	66.2	6.61 Mb	ATCC 27853
Haemophilus influenzae	Gram Neg Bacteria	38.0	1.85 Mb	ATCC 49274
Klebsiella pneumoniae	Gram Neg Bacteria	57.2	5.60 Mb	ATCC BAA-1075
Escherichia coli	Gram Neg Bacteria	50.6	5.13 Mb	ATCC 25922
Aspergillus fumigatus	Fungi	49.5	28.53 Mb	ATCC 96918
Candida albicans	Fungi	33.6	14.70 Mb	ATCC 10231
Human adenoviruses	dsDNA virus	NA	36Kb	SKLRD ADV3/GZ/0101/2011

Abbreviations: ATCC, American Type Culture Collection; NA, not available; NIFDC, National Institutes for Food and Drug Control; SKLRD, State Key Lab. of Respiratory Disease.

Table S9. Primers for microbes used for ddPCR.

No .	Species	Gene	Copy numbers	Primer	5'-3'	Length (bp)	Reference
1	Staphylococcus aureus	*tuf*	1	Forward<br>Reverse	TCCTGGTTCAATTACACCACATACTG<br>GGAAATAGAATTGTGGACGATAGTTTGA	116	[1]
2	Streptococcus pneumoniae	*lytA*	1	Forward<br>Reverse	ACGCAATCTAGCAGATGAAGCA<br>TCGTGCGTTTTAATTCCAGCT	75	[2,3]
3	Staphylococcus epidermidis	*divIVA*	1	Forward<br>Reverse	TTCCGCTCTCGTTTCCGT<br>ATTGCACGTTCTTCAGGTGT	161	[4]
4	Pseudomonas aeruginosa	*oprL*	1	Forward<br>Reverse	CAGGTCGGAGCTGTCGTACTC<br>ACCCGAACGCAGGCTATG	92	[5]
5	Haemophilus influenzae	*Hpd*	1	Forward<br>Reverse	AGATTGGAAAGAAACACAAGAAAAAGA<br>CACCATCGGCATATTTAACCACT	121	[2,3]
6	Klebsiella pneumoniae	*RcsA*	1	Forward<br>Reverse	CTATTTGCGGGTACGGAAGA<br>TCATTTGCGTTGAGATTTGC	209	[6]
7	Escherichia coli	*uidA*	1	Forward<br>Reverse	CAACGAACTGAACTGGCAGA<br>CATTACGCTGCGATGGAT	103	[7]
8	Candida albicans	*NAGl*	1	Forward<br>Reverse	TCGATATCCCGCGTGA A AAT<br>CGACCAACTCGACCTTTCTTG	201	[8]
9	Acinetobacter baumannii	*bap*	1	Forward<br>Reverse	CGCTGCAGCATCAAATCATG<br>TGGGTCAACCGAGAAAGTTACG	97	[9]
10	Aspergillus fumigatus	*FKS1*	1	Forward<br>Reverse	GCCTGGTAGTGAAGCTGAGCGT<br>CGGTGAATGTAGGCATGTTGTCC	101	This study
11	Human adenoviruses	*Hexon*	1	Forward<br>Reverse	GCCACGGTGGGGTTTCTAAACTT<br>GCCCCAGTGGTCTTACATGCACATC	132	[4]

Table S10. The scoring system for the mNGS accuracy evaluation.

Gold Standard	mNGS	TP/FN Score	TN/FP Score
Negative	Negative	NA	1 TN for all organisms not detected
Negative	Positive for 1 or more organism(s)	NA	1 FP for the organism(s) found on mNGS
Positive for 1 organism	Positive for the identical organism	1 TP for that organism	1 TN for all other organisms not detected.
Positive for 1 organism	Positive for a different organism	1 FN for organism detected by the gold standard	1 FP for different organism detected by mNGS
Positive for 2 organisms	Positive for only 1 organism	1 TP for the organism detected and 1 FN for the organism not detected.	1 TN for all other organisms not detected.
Positive for 2 or more organisms	Positive for the identical organisms	2 or (more) TP for these organisms	1 TN for all other organisms not detected.
Positive for 3 organisms	Positive for 2 of 3 organisms and positive for 2 different organisms	2 TP for the two organisms detected and 1 FN for the organism not detected	1 FP for different organisms detected by mNGS

Abbreviations: FN, false-negative; FP, false-positive; N/A, not applicable; TN, true negative; TP, true positive.

Reference

1. Loonen AJ, Jansz AR, Kreeftenberg H et al. 2011. Acceleration of the direct identification of Staphylococcus aureus versus coagulase-negative staphylococci from blood culture material: a comparison of six bacterial DNA extraction methods. Eur J Clin Microbiol Infect Dis, 30: 337-342
2. Hasan MR, Rawat A, Tang P et al. 2016. Depletion of Human DNA in Spiked Clinical Specimens for Improvement of Sensitivity of Pathogen Detection by Next-Generation Sequencing. J Clin Microbiol, 54: 919-927
3. Wang X, Theodore MJ, Mair R et al. 2012. Clinical validation of multiplex real-time PCR

assays for detection of bacterial meningitis pathogens. J Clin Microbiol, 50: 702-708
4. Mastronardi CC, Ramírez-Arcos S. 2007. Quantitative PCR for detection and discrimination of the bloodborne pathogen Staphylococcus epidermidis in platelet preparations using divIVA and icaA as target genes. Can J Microbiol, 53: 1222-1231
5. Deschaght P, Schelstraete P, Lopes dos Santos Santiago G et al. 2010. Comparison of culture and qPCR for the detection of Pseudomonas aeruginosa in not chronically infected cystic fibrosis patients. BMC Microbiol, 10: 245
6. Gupta S, Dongre A, Saxena J et al. 2017. Computation and in silico validation of a real-time PCR array for quantitative detection of pathogens isolated from blood sample in sepsis patients.
7. Chern EC, Siefring S, Paar J et al. 2011. Comparison of quantitative PCR assays for Escherichia coli targeting ribosomal RNA and single copy genes. Lett Appl Microbiol, 52: 298-306
8. Guo G. 2006. Quantification and Detection the DNA of Candida albicans in patients' blood and Antibiotic Susceptibility Research by Real-time Quantitative PCR. Peking Union Medical College:
9. De Gregorio E, Roscetto E, Iula V et al. 2015. Development of a real-time PCR assay for the rapid detection of Acinetobacter baumannii from whole blood samples. 38: 251-257
10. Piralla A, Rovida F, Baldanti F et al. 2010. Enterovirus genotype EV-104 in humans, Italy, 2008-2009. Emerg Infect Dis, 16: 1018-1021
